# Prevalence and Risk Factors for Depressive Reaction among Resident Survivors after the Tsunami following the Great East Japan Earthquake, March 11, 2011

**DOI:** 10.1371/journal.pone.0109240

**Published:** 2014-10-03

**Authors:** Chieko Matsubara, Hitoshi Murakami, Koubun Imai, Tetsuya Mizoue, Hidechika Akashi, Chiaki Miyoshi, Tamotsu Nakasa

**Affiliations:** 1 Bureau of International Medical Cooperation, National Center for Global Health and Medicine, Shinjuku-ku, Tokyo, Japan; 2 Department of Psychiatry, National Center for Global Health and Medicine, Shinjuku-ku, Tokyo, Japan; 3 Department of Epidemiology and Prevention, National Center for Global Health and Medicine, Shinjuku-ku, Tokyo, Japan; Chiba University Center for Forensic Mental Health, Japan

## Abstract

**Objectives:**

The Great East Japan Earthquake caused a gigantic tsunami which devastated coastal areas of northern Japan on 11 March 2011. Despite the large number of ‘resident survivors’ who continued to reside in their damaged houses on the second or upper floors, research on the mental health of these individuals has been limited. This study explored the prevalence of depressive reaction and risk factors for depressive reaction among these resident survivors.

**Methods:**

A cross-sectional household health support needs screening was conducted for resident survivors in Higashi-Matsushima city, Miyagi prefecture, two to four months after the tsunami. The health interview that was conducted including mental status, assessed by the Patient Health Questionnaire-2 (PHQ-2).

**Results:**

Of 5,454 respondents, 8.1% had depressive reaction. After adjustment by the number of weeks from the tsunami and the mortality rate at each respondent's place of residence, depressive reaction was significantly associated with house flooding below or above the ground floor (odds ratios of 1.92, 2.36, respectively), the unavailability of gas supply (odds ratio, 1.67), being female (odds ratio, 1.47), middle aged or elderly (odds ratios of 2.41, 2.42, respectively), regular intake of psychotropic medicine(s) since before the tsunami (odds ratio, 2.53) and the presence of one to five or more than six cohabiters (odds ratios of 0.61, 0.52, respectively).

**Conclusions:**

The results suggest a considerable psychological burden (depressive reaction) following the tsunami among resident survivors. Special supports for families with psychiatric problems need to be considered among resident survivors. Restoration of lifeline utilities and the strengthening of social ties of persons living alone may help prevent depressive reaction among resident survivors after a tsunami.

## Introduction

On the afternoon of 11 March 2011, a massive magnitude 9.0 undersea earthquake (the Great East Japan Earthquake), with an epicenter approximately 70 km off the Pacific coast, caused a gigantic tsunami that devastated the coastal areas of northern Japan. Tsunami waves reached a height of 40 m. By the end of 11 March 2013, 15,882 people had been confirmed dead, and 2,668 were missing [1]. The predominant cause of death was drowning (90.6%) [2].

The coastal city of Higashi-Matsushima in Miyagi prefecture had 65% of its urban areas engulfed by the tsunami, which travelled inland as far as 3 km from the coast. Inundation rates were highest in municipalities hit by the tsunami [3]. The city office reported that 1,061 of its 43,000 residents had died and 76% of its houses were completely or partially destroyed [3].

In the wake of the tsunami, evacuation centers were overcrowded with a large number of displaced people who accounted for up to one-third of the inhabitants [4]. However, some survivors continued to reside in their damaged houses, while some returned from the evacuation centers to their damaged homes when the floodwaters had subsided. These survivors, who often lived on the second or upper floors of their homes even though the first floor had been destroyed by the tsunami, were known as ‘resident survivors’. With distribution channels and medical institutions in the city clearly damaged and lifeline utilities cut off, the resident survivors struggled to survive on insufficient medical care and/or emergency support in their homes, many of which were almost entirely covered in mud at the ground floor, according to anecdotal reports [4].

To assess this situation, a household health support needs screening survey was conducted by the local health office in areas inundated by the tsunami, except those in which all homes had been disappeared [4]. The health interview that was conducted with resident survivors also looked at their mental status. The highest priority of this screening was to both determine depressive reaction among the resident survivors and meet their underlying needs for various types of health services. The screened survivors were then referred to regional medical services and offered health services, e.g. examination by medical doctors including psychiatric specialists, prescribed medication and/or home-care assistance.

Mental health issues are a significant concern after tsunamis, such as the one that occurred following a massive undersea earthquake along Sumatra's northwest coast in Indonesia in 2004. Researches have shown a greater prevalence of depression among displaced persons after a tsunami than non-displaced persons [5]–[7], as well as decreases in prevalence along with the time that had elapsed [5], [8]. The predictors of depression were the loss of livelihood [5], witnessing of others' suffering [9], and direct exposure to the disaster [9], [10]. Among those who were displaced, geriatrics showed unexpectedly less depression compared to non-geriatrics [11].

Previous studies have reported that social ties are linked to a lower risk of mental health problems [12]–[16]. Moreover, recent research demonstrated that social ties reduced depression symptoms following the disasters of the 1999 Chi-Chi earthquake in Taiwan [17] and the 2001 terrorist attacks in New York City [18]. In contrast, Akiyama et al. showed that perceived social ties did not significantly affect the prevalence of depression or anxiety among Burmese refugee students living in boarding houses in Thailand [19]. To date, information on the social contexts of resident survivors after a tsunami has been scant, and how social ties affect the mental health of these individuals when vital community links have been severely devastated remains largely unknown.

A better understanding of the impact of tsunamis on the mental health of resident survivors is required for designing and implementing effective public health responses that meet underlying needs for care and support. Therefore, this study aimed to explore the prevalence and correlates of depressive reaction among resident survivors after the tsunami following the Great East Japan Earthquake in 2011.

## Methods

We conducted a cross-sectional household health support needs screening.

### Study Sites

The sites targeted for the household screening included 31 local administrative areas that had been completely or partially flooded by the tsunami. In total, 7,084 registered households were targeted, and 4,672 (59.9%) households were finally confirmed to reside by the screening [4].

### Data Collection

The local health office started the household screening on April 26 and finished on July 29, 2011. Public health nurses in Higashi-Matsushima city conducted the screening, together with public health nurses from across the country who had come to assist local health officers. There was a tendency for areas with more severe destruction to be screened earlier in order to meet potentially greater demands there as rapidly as possible.

### The Target Population

The target population of the screening was composed of individuals who continued to reside in their damaged houses in tsunami-flooded areas. Because of the way in which the census was administered in the areas which were completely or partially flooded, participants whose houses were not flooded were included in the screening. People were included in the study if they resided in the targeted areas, were aged 15 years or above, and were contactable in person at home. The exclusion criteria included people who had evacuated to temporary shelters or moved outside the tsunami flooded areas, were below 15 years of age, or could not be contacted at home. Older adults who had difficulty forming answers to these questions on their own, e.g., dementia, were also excluded from this mental health screening.

Although a total of 15,503 registered residents of all ages were screened, ultimately 5,455 residents 15 years old or above were contacted in person at home. More than half of the household members were absent at the time of the survey because of work or school. Therefore, the individuals included in the screening were also likely to have been persons who were home at the time of the tsunami (Friday afternoon).

### Questionnaire

A questionnaire comprise of 11 categories that obtained demographic data such as age and gender; physical status (e.g., any chronic medical conditions, blood pressure, cough, need of nursing care); mental status, including depressive reaction and/or posttraumatic stress reactivity; and the impact of the tsunami, including flooding in the house, destruction of the house, disruption of lifeline utilities (water, electricity and/or gas), food intake frequency and direct exposure to sea water at the time of the tsunami was administered to participants. However, variables related to deceased or missing family members and lost jobs were not included in the questionnaire, as the public health nurses had determined that the survivors were reluctant to talk about such matters during the pre-visits they had conducted.

### Dependent Variable

The dependent variable in this study was “depressive reaction,” which we assessed with the Patient Health Questionnaire-2 (PHQ-2). The PHQ-2 has been validated with with general population [20], outpatients [21], college students [22], adults living with HIV/AIDS [23] and primary care [24]. The Japanese PHQ-2 was validated its sensitivity and specificity and applied into primary care [24]. It consists of the following two symptom items for major depressive episodes in the Diagnostic and Statistical Manual of Mental Disorders, Fourth Edition (the DSM-IV) [25]: “Do you feel depressed or melancholic?” and “Is there anything you used to enjoy, but are not able to now?”

We made adjustments to the response categories. The adjustments were put back to the PHQ-2's original questionnaire (PRIME-MD) [26]. Dichotomous (yes/no) rather than four-point Likert-type response sets were used in the assessment (0 = “not at all,” 1 = “several days,” 2 = “more than half the number of days,” and 3 = “nearly every day” over the last two weeks). Then, instead of using the PHQ-2 cutoff point of ≥3, we asked respondents who had ticked off both items whether these problems made it difficult for them to perform common activities of daily living, e.g. being afraid to go out after the tsunami. These adjustments were made because the public health nurses and psychiatric specialists had found that most respondents who ticked off both problems experienced these more than half the number of days over the last two weeks. This modification allowed the interviewers (the public health nurses) to identify the individuals who needed to be diagnosed and treated immediately, further ensuring the instrument's strategic practicality.

Respondents were deemed to have depressive reaction if they agreed with both items and reported difficulty performing common activities of daily living.

### Independent Variables

In the questionnaire, social tie indicators were addressed by numbers of cohabiters. Social ties are often provided by cohabiting families, communities, schools, and places of work, but the tsunami had destroyed many previously existing community links and resident survivors were now sporadically distributed across devastated areas. Therefore, the public health nurses decided not to ask questions regarding community, school, or workplace ties, which they had determined would distress the respondents. The number of cohabiters was categorised into tertile groups to explore if a bigger number of cohabiters was differently associated with the risk of depressive reaction.

The impact of the tsunami was assessed by number of weeks from the tsunami to the screening and degree of flooding in the house. Data on flooding in the house was categorised into three groups, in descending order of severity: above the ground floor, below the ground floor, and no flooding.

The availability of lifeline utilities (water, electricity and gas) was also examined to determine whether each of these was available. Behaviour and attitudes were expected to be influenced by any limited availability of lifeline utilities under post-disaster conditions. We considered the availability of water, electricity, and gas in adjusting for the impact of the tsunami on mental health.

The demographic information collected in this study included age and gender. Age was also categorized into three groups (15 to 39 years, 40 to 64 years, and 65 years or older) according to the criteria outlined by the World Health Organization (WHO) for people of middle age and the elderly [27]. We categorised age into three groups to consider the possibility of differences in risks for the middle aged and elderly compared to those who were younger.

There may have been individuals that had developed depression before the tsunami and thought it would be desirable to exclude these people from the target population. However, as the majority of medical records were lost in the tsunami, we could not identify these people through the normal means. While many of those in the target population could not remember the exact names of the medicines they had been regularly taking, most were able to recall the broad categories of their medicines (e.g., psychotropic or diabetic medicine). Therefore, in order to understand the respondents' mental health status prior to the tsunami, we asked whether respondents regularly took or were regularly taking any kinds of psychotropic medicines, such as for epilepsy or schizophrenia, or a tranquilizer or sleep inducer, since before the tsunami.

### Analysis

IBM SPSS Statistics version 21 for Windows was used to analyse the data. We conducted chi-square tests for univariate analyses. We also performed two logistic regression analyses to explore the association of the tsunami with depressive reaction using the forced-entry method, after examining multicollinearity.

Logistic regression models were conducted for depressive reaction after adjusting for number of weeks since the tsunami and the mortality rate at each respondent's place of residence [28]. The morality rate was applied into the regression model to consider the prevalence of depression in a residential community with numbers of casualties, as we avoid harming the respondents emotionally by asking any items regarding deceased or missing family members at that point in time.

*P*-values of ≤0.05 were considered to be statistically significant.

### Ethics Statement

All participants were informed about the objectives, the screening procedures, the possibility that the results would be utilized for a study, and the confidentiality of the study, with the aid of a prepared information sheet. The participants were explained an opportunity to refuse inclusion at any time during the screening. Verbal consent was obtained prior to enrolment, although the Ethical Guidelines for noninterventional epidemiological studies without human specimen collection does not require informed consent stipulated by the Ministry of Education, Culture, Sports, Science and Technology and the Ministry of Health, Welfare, Labour and Welfare, Japan. Written consent was not obtained, as it was not the requirement for noninterventional epidemiological studies without human specimen collection. For subjects aged below 18 years, we conducted the screening after obtaining parental agreement and that of their children. We prepared and retained a name list of the participants who agreed to participate in the screening. All household screening protocols and procedures, including the informed consent procedure, were reviewed and approved by the Research Ethics Committee of the National Center for Global Health and Medicine, Japan (Ethics approval code: NCGM-G-001103-00). The National Center for Global Health and Medicine acted in collaboration with the city of Higashi-Matsushima with regard to the restoration of public health. A total of 87 household members declined to participate in the screening. Numerical codes were used in place of names at the time of analysis to ensure confidentiality.

## Results

Table 1 lists the univariate analyses for depressive reaction. Among the 5454 respondents, 10.0% respondents ticked off both items of the PHQ-2 and 8.1% of them had difficulties in daily common activities, regarded as depressive reaction. There were more respondents who were female (67.2%) and aged 65 years or older (39.6%) than in the city's population. The houses of more than 80% of the respondents had been inundated by the tsunami at the screening areas. The mean number of weeks from the tsunami was 12.7 weeks (Standard Deviation, 2.9). Covariates associate with depressive reaction were gender (*p* = 0.009), age (*p*<0.001), regular taking of psychotropic medicine(s) since before the tsunami (*p*<0.001), the height of house flooding (*p*<0.001), the number of cohabiters (*p* = 0.012) and the unavailability of gas supply (*p* = 0.002).

**Table 1:** Univariate analysis of depressive reactivity in various variables among the resident survivors after the tsunami.

		Number of respondents†	Depressive reactivity	
			%	*p*-value‡
Total		5454	8.1	
The mortality rate at the place of residence	0 to 0.50%	1724	7.9	0.631
	0.51 to 1.0%	1671	7.7	
	1.1 to 5.0%	1212	8.7	
	5.1 to 10.0%	0	0	
	10.1% and over	129	10.1	
Gender	Male	1790	6.6	**0.009**
	Female	3664	8.8	
Age	15 to 39 years old	947	3.3	**<0.001**
	40 to 64 years old	2347	9.2	
	65 years old and over	2160	9.1	
Direct exposure to sea water	Yes	597	9.6	0.196
	No	4742	7.9	
Regular intake of psychotropic medicine(s) since before the tsunami	Yes	176	3.2	**<0.001**
	No	5278	96.8	
				
House flooding	Above the ground floor	3418	9.6	**<0.001**
	Below the ground floor	673	8.0	
	No flooding	927	4.0	
The number of cohabiters	More than 6 persons	296	8.1	**0.012**
	1 to 6 person(s)	4632	7.7	
	None	526	11.7	
Water supply	Yes	4224	8.3	0.488
	No	152	9.9	
Electricity supply	Yes	4252	8.3	0.130
	No	95	12.6	
Gas supply	Yes	4035	8.0	**0.002**
	No	231	13.9	

† : Number may not be add up to 5454 because not all the respondents answered all the questions.

‡ : Chi-square test.

Table 2 shows the results of the multivariate regression analysis for depressive reaction. After adjusting for number of weeks from the tsunami and the mortality rate at each respondent's place of residence, we found that depressive reaction was significantly associated with being female (odds ratio, 1.47), middle aged or elderly (odds ratios of 2.41 and 2.42, respectively), regular taking psychotropic medicine(s) since before the tsunami (odds ratio, 2.53), house flooding below or above the ground floor (odds ratios of 1.92 and 2.36, respectively), the presence of 1 to 6 or more than 6 cohabiters (odds ratios, 0.61, 0.50, respectively), and the unavailability of gas supply (odds ratio, 1.67).

**Table 2:** Logistic regression analysis for depressive reactivity using forced-entry method.

		Depressive reactivity (n = 4634)				
		B	S.E.	*p*-value	OR	95%CI
Week		−0.05	0.02	**0.014**	0.95	(0.91–0.99)
The mortality rate at the the place of residence	0 to 0.5%	Ref.				
	More than 0.5 to 1.0%	0.08	0.14	0.578	1.08	(0.82–1.43)
	More than 1.0 to 5.0%	−0.02	0.15	0.894	0.98	(0.73–1.32)
	10.0% and over	0.57	0.43	0.178	1.78	(0.77–4.09)
Gender	Male	Ref.				
	Female	0.39	0.13	**0.003**	1.47	(1.14–1.90)
Age	15 to 39	Ref.				
	40 to 64	0.88	0.22	**<0.001**	2.41	(1.56–3.72)
	65+	0.88	0.22	**<0.001**	2.42	(1.56–3.76)
Regular intake of psychotropic medicine since before the tsunami	No	Ref.				
	Yes	0.93	0.23	**<0.001**	2.53	(1.62–3.97)
						
House flooding	No	Ref.				
	Below the ground floor	0.64	0.25	**0.010**	1.92	(1.17–3.10)
	Above the ground floor	0.86	0.21	**<0.001**	2.36	(1.57–3.54)
The number of cohabiter(s)	None	Ref.				
	1 to 6 person(s)	−0.50	0.17	**0.003**	0.61	(0.44–0.85)
	More than 6 persons	−0.65	0.33	**0.050**	0.52	(0.28–1.00)
Direct exposure to sea water	No	Ref.				
	Yes	0.06	0.17	0.750	1.06	(0.75–1.49)
Water supply	Yes	Ref.				
	No	0.15	0.37	0.676	1.17	(0.56–2.42)
Electricity supply	Yes	Ref.				
	No	0.16	0.37	0.676	1.17	(0.56–2.41)
Gas supply	Yes	Ref.				
	No	0.51	0.25	**0.038**	1.67	(1.03–2.71)
Cox-Snell R^2^		0.02				
Nagelkerke R^2^		0.06				

## Discussion

The results of this study demonstrated that 8.1% of the resident survivors had depressive reaction from two to four months after the 11 March tsunami. To our knowledge, this is the first study to report on depressive reaction among resident survivors after a tsunami.

The results showed a much higher prevalence of depressive reaction among respondents than in the general population (as shown in the benchmark World Mental Health Japan Survey 2002–2003 with the DSM-IV). That study showed that, within the previous year, the prevalence rates of depression were 2.9% (95% CI = 2.1, 3.7) [29].

The prevalence of depressive reaction decreased along with the increase in the number of weeks from the tsunami. These results were consistent with previous studies [5], [8], [30], and suggested that depressive reaction would diminish over time.

The height of house flooding was a significant predictor of depressive reaction. The result suggested that the level of house damage would reflect the level of distress among resident survivors, who continued to live in their damaged houses. Since it is common for Japanese to put off their shoes in the house, the resident survivors whose houses were flooded above the ground floor would be inconvenient in their houses. Even if the distance from the coast was the same, the level of flooding in the house could be different depending on the landscape. Therefore, the data obtained through the household screening could provide substantial information on levels of flooding in houses, which would reflect the eventual impact of the tsunami more accurately.

However, direct exposure to sea water at the time of the tsunami was not associated with depressive reaction in this study. Future studies to be conducted if an approach can be found to assess the height of exposure to sea water with less burdensome to respondents.

We found a significant association between the female gender and depressive reaction among the resident survivors. These results were consistent with the results of most previous reports [18], [31]–[33].

A higher risk was found for depressive reaction among persons aged 40 to 64 years and 65 years or older than those aged 15 to 39 years. Approximately 140,000 to 200,000 persons were estimated to have lost their jobs as a result of the Great East Japan Earthquake [34], and many of them were residents in the devastated coastal areas [35]. This lost job might be related to a burden for those who lost their jobs because of the tsunami, as shown in a previous study conducted after a terrorist attack [18].

A significant association was found between depressive reaction and regular intake of psychotropic medicine(s) since before the tsunami. We used “regular intake of psychotropic medicine since before the tsunami” instead of “developed depression since before the tsunami”, as we could not identify persons who had developed depression before the tsunami by medical records. Given that persons engaged in “regular intake of psychotropic medicine since before the tsunami” included those with depression, a significant association between “depressive reaction” and “regular intake of psychotropic medicine since before the tsunami” would be expected.

According to anecdotal reports, some resident survivors had hoped to continue staying at the evacuation centers but could not due to conditions their family members had, such as autism and schizophrenia. Therefore, it would be necessary to consider special support for such families coping with psychiatric problems to further ensure the efficacy of public health programs geared toward survivors of the tsunami.

The results of this study offer insight into the protective effects of social ties provided by cohabiters in terms of the incidence of depressive reaction among resident survivors. Associations between weaker social ties and the development of depression have been also reported after a terrorist attack [18] and an aircraft accident [36].

The effect exerted by the presence of cohabiters might be due to the following two reasons. First, the cohabiters might play an important role in allowing the survivors to share their traumatic experiences, and thus provide critically needed emotional support in the form of person-to-person contact. It is reasonable to speculate that cohabiters helped mitigate the sense of loneliness and isolation felt by resident survivors through their reactions in the aftermath of the crisis. As mentioned earlier, the resident survivors exhibited noticeable difficulties in retelling their experiences at the time of screening. Anecdotal reports by international counsellors, who had immediately been dispatched to the scene after the tsunami, also described how profound their sense of helplessness was, and the survivors' inability to talk about what had happened or how they felt.

Second, cohabiters could have shared crucial information on the social context with each other in the post-disaster situation. Given the circumstances, this information is potentially more critical for resident survivors than for refugees at the evacuation centers. This is because the tsunami destroyed their community links and communication networks which had existed previously; therefore, support depended to a large extent on the resources available via social ties. For example, food supply and/or medical services were mainly available at the evacuation centers, and resident survivors needed to know the schedules for these services as well as their locations. Cohabiters, even children, can help obtain more information and establish more dense social networks [37]. Thus, cohabiters would be expected to play important roles in the formation of rudimentary social ties and networks after a catastrophe such as a tsunami.

Further, the characteristics of social ties of residents of Miyagi prefecture may have worked supportively after the tsunami. In general, the people living in Miyagi tend to participate more frequently in civic activities such as volunteer work and be associated with people at school or the office, but perform fewer activities with others in the neighbourhood or society [38]. As a result, the information brought by cohabiters from across the residential community would take on an even greater importance for the resident survivors.

Finally, the prevalence of depressive reaction was also associated with the disruption of lifeline utilities, particularly gas supply. As of 31 March 2011, the rate of restoration was more than 90% for electricity and 70% for water, but only 30% for gas in Miyagi prefecture [39]. Even though most households in Higashi-Matsushima city were provided with liquefied petroleum gas, the gas supply was only restarted two weeks after the tsunami. This was because an electrical power outage after the tsunami disturbed the gas filling instruments [40]. This disruption of the gas supply exerted a major impact on the lives of the victims in terms of their cooking, bathing, and heating, all of which are closely related to the quality of life in a wintry region. Anecdotes confirmed that efforts to restore lifeline utilities post-disaster encouraged the resident survivors who experienced prolonged difficulties in their lives after the tsunami.

In conducting this study, we noted several limitations which may have had an effect on the scope and generalizability of the results. First, a greater percentage of elderly women were included in this study than that in the city's population. These elderly female residents were also more likely to have been affected by the tsunami while at home, as the earthquake happened at 14:46. Second, the screening was conducted earlier in areas where destruction was greater, so the regression analysis was adjusted for the number of weeks since the tsunami. Third, the questionnaire did not include any variables about deceased or missing family members, lost jobs because of the tsunami, or other variables related to the survivors' social networks other than the number of cohabiters, or variables related to peritraumatic distress [41], because it was apparent in the pre-interviews that the respondents were reluctant to answer such questions. It would be a probable reason why the regression model in this study did not explain depressive reaction well, even though we applied the mortality rate at the each respondent's place of residence and did not found significant association between the depressive reactivity and the mortality rate. In previous papers, Adams et al. has explained depression with an R square of 0.24 using a regression model that included perievent panic after the terrorist attacks in New York City [42], and Akiyama et al. depression with an R square of 0.23 using a regression model that included traumatic events distribution among Burmese refugee students [19]. Future studies that include these variables are needed if an approach can be found to make such inquiries less burdensome. Fourth, this screening could not exclude respondents who have depressive problem before the tsunami and who have mild cognitive impairment as with previous studies.

## Conclusions

The results suggest that there is a considerable psychological burden (depressive reaction) among resident survivors after a tsunami. The predictors of depressive reaction were being female, middle-aged or elderly, regular intake of psychotropic medicine(s) since before the tsunami, house flooding (especially above the ground floor), an unavailability of gas supply, and living alone. Special supports for families with psychiatric problems need to be considered among resident survivors. Strengthening of social ties for persons living alone and restoration of lifeline utilities to their normal state would ameliorate the development of depression among resident survivors who continue to reside in their homes in the aftermath of a tsunami.
